# Assessment of digital therapeutics in decentralized clinical trials: A scoping review

**DOI:** 10.1371/journal.pdig.0000905

**Published:** 2025-06-23

**Authors:** Cinja Koller, Marc Blanchard, Thomas Hügle

**Affiliations:** Department of Rheumatology, Lausanne University Hospital and University of Lausanne, Lausanne, Switzerland; The University of Hong Kong, HONG KONG

## Abstract

This scoping review aims to identify the necessary and practical considerations for the design, conduct and safety of decentralized clinical trials (DCTs) that test digital therapeutics (DTx) or software as a medical device (SaMD). The review follows the framework of Arksey & O’Malley. A search strategy with the keywords “Digital therapeutics” or “Software as Medical Device” AND “decentralized clinical trial” or synonyms was applied to Cochrane CENTRAL, EMBASE, MEDLINE and Web of Science databases with the latest search on the 25th of April 2025. We selected peer-reviewed articles reporting about fully or partly DCTs using apps or devices that were classified as DTx or SaMD. Studies using general health software or not focusing on the design or experiences of the DCT were excluded. Main study characteristics were extracted and the articles thematically coded with the qualitative software Atlas.ti. 335 results were assessed for title and abstract screening and 113 articles were identified for full-text screening, of those 41 fulfilled inclusion criteria. DTx used in the trials were mainly targeting depression. The clinical trial design differed significantly in the number of study arms (1–16), participants (11─5602) and blinding. E-recruitment (78%), e-eligibility screening (73%), e-informed consent (68%), inclusion of electronic-patient reported outcomes (e-PROs) (88%), passive data collection (59%) and use of reminders (59%) were key reoccurring features of the studies. Effective access and inclusion of participants, but low adherence and engagement is highlighted in most studies. In some cases, only 40% of participants installed the app and significant drop-out rates of about 50% are reported. A framework for DCTs evaluating DTx is provided. In summary, DCTs for DTx are unstandardized, heterogenous and characterized by low adherence. Further research on how to tackle the engagement problem, along with clearer guidance and regulatory frameworks, is required to standardize this trial type in the future.

## Author summary

We systematically reviewed the literature and analyzed 41 articles that investigated digital therapeutics (DTx) or a software as a medical device (SaMD) with a decentralized clinical trial (DCT) or its benefits and challenges. Characteristics such as trial design, health area under investigation, methodology or outcomes were summarized and compared. Although trials varied, overlapping themes between the included articles were identified, namely e-recruitment, e-eligibility screening, e-consent, patient-reported outcomes, reminders and passive data collection. Based on these themes, we provided a framework for DCTs testing DTx to standardize future trials. The main advantages reported in the included studies were better reach, access and faster recruitment than with traditional trials, often resulting in diverse participant samples. The predominant mentioned difficulty of this trial type was engagement with the DTx. Therefore, more research is needed to develop strategies to address this problem.

## Introduction

In the rapidly evolving landscape of digital health, digital therapeutics (DTx) are being frequently used and developed, especially for self-management in chronic diseases. Typically, they provide information, monitor patient reported outcomes (PROs), and often incorporate psychological and behavioral therapy approaches [1,2]. DTx are highly accessible, given that a majority of people use the internet and own a mobile phone. Moreover, they can easily be personalized with a patient-centered approach [2,3]. The term DTx is defined by the Digital Therapeutics Alliance [4] as: “Digital therapeutics deliver to patients evidence-based therapeutic interventions that are driven by high quality software programs to treat, manage, or prevent a disease or disorder. They are used independently or in concert with medications, devices, or other therapies to optimize patient care and health outcomes.”.

In the United States (U.S.), DTx fall under the rules and concepts of software as a medical device (SaMD) [5,6]. The International Medical Device Regulators Forum defines SaMD as software designed for one or more medical purposes, capable of fulfilling these purposes independently of a hardware medical device [7]. While in the U.S. all DTx qualify as SaMD, this does not apply vice versa, as they do not necessarily deliver a therapeutic intervention to the patient. For example, rapid large vessel occlusion (LVO), a Food and Drug Administration cleared software for identification of suspected large vessel occlusions [8], is used by physicians to help with decision making but is not used by patients themselves as a therapeutic intervention.

The distinction between a DTx and other educational health or wellness apps can be made through the clinical evaluation [2]. A DTx is designed to have a meaningful effect on treatment or management of a specific disease, rather than providing purely informational content, and must therefore undergo clinical evaluation to ensure its effectiveness and safety [2,4,6,9]. After demonstrating safety and efficacy in a clinical trial, a DTx can seek regulatory approval—such as registration as a DiGA (Digitale Gesundheitsanwendungen) in Germany—and may be reimbursed by health insurers in an increasing number of countries [4,10].

However, the clinical trial process that underpins this regulatory pathway is often resource-intensive, time-consuming, and limited in reach. Traditional controlled clinical trials are characterized by substantial financial and time expenditures, restricted access, a poor retention rate and limited generalizability of results [11,12]. The COVID-19 pandemic accelerated the use of digital technologies in healthcare and provoked a shift in the way clinical trials are conducted [13]. Major changes in clinical trials are reflected in increased online recruitment, adoption of electronical consent, the shipment of samples or devices to and from the patients home and telemedicine consultations [1,13,14].

These changes are particularly relevant when discussing decentralized clinical trials (DCTs). The Clinical Trials Transformation Initiative defines DCTs as “those in which some or all study assessments or visits are conducted at locations other than the investigator site via any or all of the following DCT elements: tele-visits; mobile or local healthcare providers, including local labs and imaging centers; and home delivery of investigational products.” [15]. DCTs can be completely remote or partially decentralized with hybrid approaches via study centers. The latter requires some visits on site, while other visits or assessments can be performed at a home or within a local care community. In fully remote trials, patients have no required site visits [15]. Existing evidence on DCTs is diverse, with studies focusing on different technologies and evaluation methods. Much of the literature explores specific digital elements used in DCTs, such as e-recruitment and e-consent; however, challenges related to data privacy, accuracy, and integration into existing healthcare infrastructures remain insufficiently addressed [1]. DCTs have been proposed to accelerate patient recruitment, broaden participant diversity by overcoming geographical distance, improve patient retention due to a lower participation burden, decrease the costs of the trial and produce data which is much closer to the real-world [3,11,13,16–18]. A systematic review on DCT methods found that there is insufficient evidence to establish a best practice method, and the experiences of staff and participants are underrepresented [3].

In this context, DTx are particularly well suited for DCTs as their evaluation ideally involves a randomized clinical trial (RCT). Since DTx are used in remote settings, their evaluation with a DCT is closer to the real-world use than with a traditional trial, which could potentially improve the applicability of the results. Furthermore, they often provide the user interface for the collection of PROs and the therapeutic intervention, making them inherently compatible with remote data collection. DTx also typically require fewer interactions with healthcare providers and do not bring along complex supply chain management issues [13,19]. DCT and DTx are both facilitating access by bringing the research or the treatment to the patients home rather than the other way around [2,15]. Lastly, DTx trials in remote settings appear to be safe, with minimal risk of serious adverse events compared to pharmacological interventions [2]. Together, these factors make DCTs an appropriate approach for evaluating DTx.

While various countries and entities have issued guidance on DCTs, these recommendations are not specifically tailored for DTx trials [20–22]. Research on DTx trials has examined aspects such as design, quality, and evaluation. DTx evaluation is resembling drug trials and increasingly incorporates digital biomarkers and real-world data [23]. Studies have noted inconsistencies in the definition of control conditions and recommended the use of large-scale, real-world effectiveness trials [6]. Despite these identified biases in the evaluation, most DTx efficacy studies were designed as RCTs [10]. A recent systematic review characterizing DTx trials revealed that they are typically short in duration and, like drug trials, tend to lack inclusivity in terms of participant diversity and eligibility criteria [24].

However, no systematic or scoping review focusing on the combination of DCTs with DTx has been identified, prompting the need for this review to fill this research gap. Therefore, this scoping review aims to identify insights from existing literature and practice on the use of DCT methods in evaluating DTx. The objectives are to address the key aspects and practical considerations when planning, designing, conducting or monitoring DCTs with DTx or SaMD, summarize current knowledge and challenges faced during these trials. Moreover, as running a DCT with DTx is still in its infancy, the article aims to identify research gaps, the type of evidence currently available and what trends, key terms or concepts are used in the literature.

## Methodology

The scoping review follows the framework for scoping reviews provided by Arksey and O’Malley [25] and Levac et al. [26], and is structured along the PRISMA-ScR checklist [27]. The literature research was guided by a structured search strategy. Different terms for DCTs (“virtual”, “remote”, “end-to-end”, “web-based”, “ehealth”) were included based on the systematic review on DCTs conducted by Rogers et al. [3]. The term “mHealth” (mobile health), was included as the term “DTx”, as defined by the DTx Alliance is relatively new. This could have led to the exclusion of articles using other terms that would fall under the definition of a DTx or SaMD. Moreover, many DTx come in form of an mHealth application. The protocol and PRISMA-ScR checklist for this scoping review can be accessed under Supporting information (S1 and S2 Files).

### Inclusion criteria

Fully or partially DCTs, investigating DTx/SaMDs with intention to treat, manage or prevent a disease as per definition of the DTx Alliance, unrelated to planned regulatory approval are included in the review. Also, the protocols, trial design reports or articles reporting experiences about DCTs or a part of it evaluating a DTx/SaMD are considered for inclusion. A fully DCT requires no in-person visit by the patient at the research center, the clinic or a pharmacy while a partially DCT includes at least one physical visit of participants.

### Exclusion criteria

**Fig 1:**
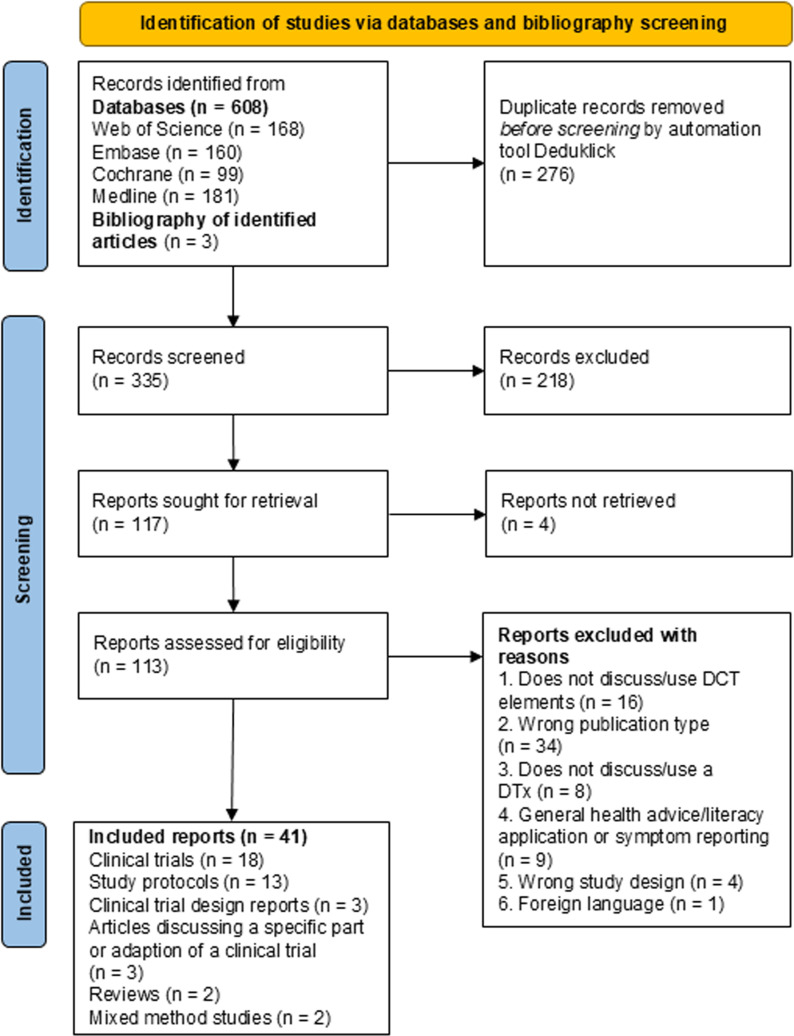
Selection process of literature – PRISMA flowchart.

Articles that are not focused on the design, planning, conduct or monitoring or its related learnings (difficulties, benefits and advantages compared to classical trials) of the DCT were not included in this review (reason 1, Fig 1). Only peer-reviewed articles were considered for inclusion whereas abstracts were excluded (reason 2, Fig 1). If the DTx of interest was a wellness or well-being app or a device that is not focused on the treatment, management or prevention of a disease or if the use of the DTx was not discussed, the article was not considered (reason 3, Fig 1). Studies were excluded if the software did not have a specific medical purpose, but instead focused on general health advice, health literacy or symptom reporting (reason 4, Fig 1). Articles with study designs other than clinical trials, for example cross-sectional studies were not considered either (reason 5, Fig 1). Full texts not available in English, French or German were excluded (reason 6, Fig 1).

### Databases and search strategy

For the identification of peer-reviewed articles, the databases Web of Science, MEDLINE, EMBASE and Cochrane CENTRAL were searched with a structured search strategy which was composed together with a librarian. The last search was conducted on 25^th^ of April 2025. The search strategy, with the Boolean operators OR and AND, was applied in the four databases. The following presentation of the search strategy was used in EMBASE:

((DCT OR DCTs OR VCT OR VCTs OR (trial* NEAR/3 (decentrali* OR de-centrali* OR remote* OR virtual* OR web-based OR ehealth OR end-to-end OR hybrid))):ab,ti,kw) AND (‘mobile health application’/exp OR (“digital therapeutic*” OR DTx OR SAMD* OR (software NEAR/5 “medical device*”) OR Mhealth OR DIGA OR “digital health application*” OR “mobile health” OR (“mobile app*” NEAR/3 treat*)):ab,ti,kw)

No limitations such as language restrictions or year of publication were used. Additionally, the references of the articles found with the systematic search strategy were screened for matching articles.

### Screening process and data extraction

After applying the search strategy to the four chosen databases, the 608 results were imported in EndNote 20 and deduplicated with Deduklick, which led to 332 results [28]. The remaining articles were imported in Rayyan for eligibility screening [29]. 3 articles were found by bibliography screening of the included articles. Articles were first included or excluded based on abstract screening by two reviewers (CK & MB). Afterwards full text screening was done by the same two reviewers using beforehand defined eligibility criteria. In case of disagreement or uncertainty for in- or exclusion of articles the reviewers resolved the issue by discussion. During the abstract screening no conflicts occurred but 7 articles were labeled as “maybe” and then resolved by consensus. Round two based on full text screening led to (10/113) disagreements and (7/113) uncertainty. Data extraction was done by two reviewers based on the articles and the information provided in the trial’s registry.

### Analysis of selected literature

**Fig 2:**
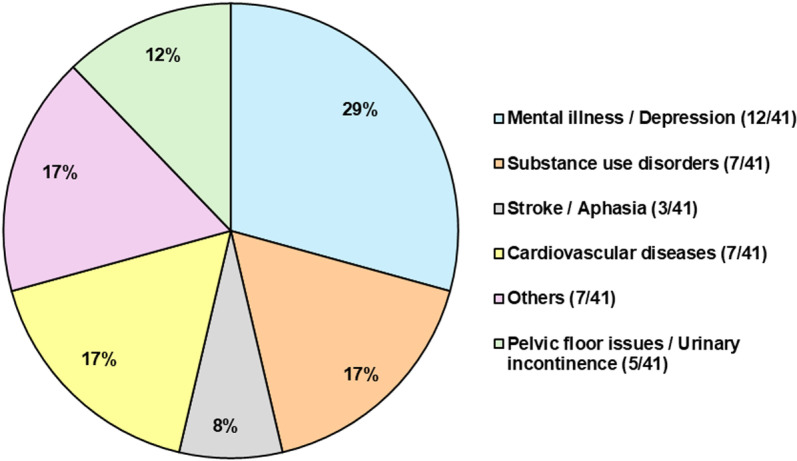
Therapeutic indications of DTx or SaMD investigated in the articles.

As suggested by Levac et al. [26], the selected literature was reviewed with qualitative coding to identify reoccurring themes across the articles. Two rounds of coding by one researcher with the software Atlas.ti, resulted in 42 codes [30]. Coding was oriented towards the research question with predefined code groups (inductive coding) but went beyond the topics of design, planning, conducting and monitoring (deductive coding). The emerging codes were grouped into five prospectively defined code groups, namely design, planning, conducting, challenges and benefits. The five code groups were selected by two researchers. After one round of coding, the same two researchers discussed new codes and text sections that were coded with the predefined code groups, to ensure consensus and that they followed the intended scope before proceeding to the second round. Some codes remained without being assigned to a group as a semi-structured approach was used. Articles were grouped by disease or condition targeted and were reviewed along key aspects of the studies and the data was charted in a Microsoft Excel spreadsheet. Results of the Excel were summarized and illustrated in Table 1 and Fig 2. When using the term DCT, the study refers to the definition of the DCTs working group from the Clinical Trials Transformation Initiative and for the term DTx to the DTx Alliance definition.

**Table 1:** Summary of key data of included studies.

Author(s), year of publication, study location, Citation	Type of article Targeted disease/condition	Intervention type, and comparator (if any); duration of the intervention	Study populations (carer group; care recipient group) Number of participants	Methodology Study design: Fully remote or hybrid	Aims of the study	Outcome measures	Important results
Schweiger et al. 2022 Missouri, U.S. [31]	Protocol Depression	1^st^ Arm (Experimental): Device: Mindful My Way No comparator 18 weeks	Older adults (65+) with current major depression N = 23	Single arm, open-label early-phase clinical trial Fully remote	Test online mindfulness training	Ecological Momentary Assessment (EMA) Depression Scale	Exemplifies innovative clinical trial designs by using smartphone technology
Pratap et al. 2018 California, U.S. [46]	Clinical trial report Depression	1^st^ Arm (Active Comparator): Therapeutic video game targeting cognitive control network 2^nd^ Arm (Experimental): Problem Solving Therapy 3^rd^ Arm (Active comparator): Basic health push app 4 weeks	People 18 and older who have symptoms of depression Enrolled: N = 1040 Active users: N = 348	Feasibility pilot study randomized, open-label clinical trial Fully remote	Feasibility of conducting future randomized controlled trial comparing three mobile mental health apps for depression	Sheehan Disability Assessment Scale	Long-term engagement a key issue. Improved outcomes did not vary by treatment app
Arean et al. 2016 San Francisco, U.S. [47]	Clinical trial report Depression	1^st^ Arm: Behavioral: Problem-solving therapy 2^nd^ Arm: Behavioral: Case management (CM) and 3^rd^ Arm: control app for mood 12 weeks	People older 60 suffering from major unipolar depression N = 626	Open-label randomized controlled trial Fully remote	Compare use patterns and clinical outcomes between 3 different self-guided mobile apps for depression	Depression [Time Frame: Measured at pretreatment and Weeks 3, 6, 9, 12, and 24]	Depression apps have greatest impact on people with more moderate levels of depression
Anguera et al. 2016 San Francisco, U.S. [48]	Clinical trial report Depression	1^st^ Arm: A cognitive training application, based on problem-solving therapy. 2^nd^ Arm: Control app for mood 12 weeks	People older 60 suffering from major unipolar depression N = 1098	Open-label randomized controlled trial Fully remote	Assess feasibility fully mobile randomized controlled trial using assessments and treatments delivered through mobile devices	Access (sample representativeness), engagement and costs to complete study	Study engagement high during the first 2 weeks of treatment, falling to 44% adherence by the 4th week
Furman et al. 2023 San Francisco, U.S. [34]	Protocol Depression	1^st^ Arm (Experimental): Active intervention 5 weeks app program 2^nd^ Arm (Control): No intervention, usual care 5 weeks	Participants aged 13–21 with symptoms of depression N = 220	2 Arm randomized, parallel assigned, single-blind trial Fully remote	Evaluate the effectiveness of a self-guided, cognitive behavioral therapy (CBT)-based mobile app + assessment-enhanced Usual Care (eUC) compared to eUC alone as an intervention	Depressive symptom severity at post-intervention	Not applicable
McCloud et al. 2020 United Kingdom [55]	Clinical trial report Depression	1^st^ Arm (Experimental): Feel stress free mobile application 2^nd^ Arm (Control): No intervention, waitlist 12 weeks	Students (18+) with anxiety or depressive symptoms N = 168	2 Arm parallel-assigned randomized open-label trial Fully remote	Test the effectiveness of an application-based computerized CBT intervention named “Feel Stress Free” at reducing depression and anxiety symptoms in a sample of UK university students	Hospital Anxiety and Depression Scale	Feel Stress Free app reduced depression symptoms at week 6
Kulikov et al. 2023 San Francisco, U.S. [51]	Clinical trial report Depression	1^st^ Arm (Experimental): Limbix Spark, CBT-based intervention 2^nd^ Arm (Active Comparator): Psychoeducation 5 weeks	Adolescents with depression age 13–21 N = 60	2 Arm randomized, parallel-assigned, open-label trial Fully remote	Evaluate the clinical effectiveness and safety of a CBT- based digital treatment for adolescent depression (Limbix Spark) relative to psychoeducation)	Change in depression symptoms, Participant-/parent-rated Anxiety Symptoms and Global Functioning, number of participants in remission	Robustness of online recruitment techniques, strong engagement with and potential therapeutic benefit of Spark, and effectiveness of the novel safety protocol to monitor and ensure patient safety. Significant main effect of time, associated with decreased PHQ-8 scores over time
Moberg et al. 2019 San Francisco, U.S. [56]	Clinical trial report Depression	1^st^ Arm (Intervention): Mobile application Pacifica (CBT) + treatment as usual 2^nd^ Arm (Control): No intervention, waitlist + treatment as usual 4 weeks	Adults (18+) with mild to moderate symptoms of depression or anxiety N = 500	2 Arm, factorial assigned open-label trial Fully remote	Examine the efficacy of a mobile application implementation of existing best practices in mental health treatment for managing stress, anxiety, and depression	Change in Depression, Anxiety, Stress [Time Frame: 2 weeks, 1 month, 3 months]	Pacifica group experienced significant change from pre to post for each variable. Change in the Pacifica group was greater than change in the waitlist group
Rothman et al. 2024 Texas, U.S. [42]	Protocol Depression	1^st^ Arm: App-based DTx (CT-152), cognitive-emotional and behavioral intervention 2^nd^ Arm: Sham Dtx 6 weeks	Adults with major depressive disorder aged 22–64 years N = 386	2 Arm, phase 3 randomized, parallel-assigned, blinded and controlled trial Hybrid	Compare the effectiveness of 2 DTx in adults diagnosed with major depressive disorder who are on antidepressant therapy	Change from baseline to week 6 in the Montgomery–Åsberg Depression Rating Scale and General Anxiety Disorder 7 total score	Methodologically robust trial, incorporating many aspects of a conventional pharmaceutical phase 3 trial
Akechi et al. 2024 Japan [61]	Clinical trial report Depressive symptoms	1^st^ Arm: App with psychoeducation, assertive training, problem-solving, behavioral activation 2^nd^ -16^th^ Arm: App with psychoeducation + all different combinations of the three components in Arm 1 8 weeks	Adults (20+) with cancer diagnosis N = 359	Parallel-group, randomized, open-label, factorial trial Fully remote	Develop an efficient and effective smartphone psychotherapy component to address depressive symptoms	Change in patient health questionnaire-9 and Generalized Anxiety Disorder 7 between baseline and week 8.	Significant reduction of depressive symptoms but none of the three app intervention components contributed to additive significant reduction of depressive symptoms
Haun et al. 2023 U.S. [70]	Report of recruitment and attrition in a clinical trial Posttraumatic Stress Disorder (PTSD)/ Musculoskeletal Chronic Pain	1^st^ Arm (Active Comparator): Mission Reconnect self-directed mobile app intervention that teaches complementary and integrative health skills. 2^nd^ Arm (Placebo control): Waitlist 8 weeks	Veterans with musculoskeletal chronic pain or PTSD N = 364	2 Arm randomized, single group assignment, waitlist controlled, open-label trial Hybrid	Describe the recruitment, onboarding phase, and attrition of a fully remote randomized controlled trial assessing the efficacy of a self-directed mobile and web-based intervention for veterans with PTSD and their partners.	Mission Reconnect effectiveness for physical, PTSD, and psychological symptoms, global health	Of the 364 recruited dyads, 97 (26.6%) failed to complete onboarding activities. Reasons for failure include loss of self-elected partner buy-in (n = 8, 8%), difficulties with using remote data collection methods and interventions (n = 30, 31%), and adverse health experiences unrelated to study activities (n = 23, 24%)
Ben-Zeev et al. 2021 Washington, U.S. [53]	Clinical trial report Serious mental illness	1^st^ Arm (Experimental): CORE smartphone app 2^nd^ Arm (Active Comparator): Waitlist control 4 weeks	Adults (18+) diagnosed with a serious mental illness N = 315	2 Arm randomized waitlist-controlled, crossover, open-label trial Fully remote	Evaluate acceptability and preliminary efficacy of CORE, a smartphone intervention that comprises daily exercises designed to promote reassessment of dysfunctional beliefs	Change in depressive symptoms, paranoid thinking, anxiety symptoms, psychotic symptoms and participant acceptability (after 1 and 2 months)	Feasibility of remote digital health app trial. Tested CORE app is usable, acceptable, effective for reducing symptoms in people with serious mental illness
Park et al. 2022 U.S. [39]	Protocol Alcohol use disorders	1^st^ Arm (Active Comparator): Self-monitored smartphone app A-CHESS 2^nd^ Arm (Experimental): Peer supported smartphone app A-CHESS 3^rd^ Arm (Experimental): Clinically integrated smartphone app A-CHESS 12 weeks	Adults aged >21 years with risky drinking pattern N = 357, enrollment suspended due to COIVD-19	3 Arm randomized, parallel-assigned, open-label trial Hybrid	Detect the effectiveness of an mHealth intervention by assessing differences in self-reported risky drinking patterns and quality of life between participants in three study groups (self-monitored, peer-supported, and clinically integrated)	Number of risky drinking days (1year)	Not applicable
Kaufman et al. 2023 U.S. [62]	Clinical trial design report Fetal Alcohol Spectrum Disorder Prevention	1^st^ Arm (Experimental): mHealth intervention WYSE CHOICES prevention program 2^nd^ Arm (Control): No intervention, unrelated program to alcohol exposed pregnancy (AEP) prevention 3 hours	American Indian and Alaska Native (AIAN) Women age 16–20 years	2 Arm randomized, parallel-assigned, open-label trial Fully remote	Test the effectiveness of a culturally adapted mHealth intervention to prevent AEP, using social media to recruit AIAN young women	Number of days in past 30 days with at least one alcoholic drink, effective contraceptive use	Not applicable
Kaufman et al. 2025 U.S. [60]	Clinical trial report (one month outcomes) Fetal Alcohol Spectrum Disorder/ Alcohol-exposed pregnancy	1st Arm (Experimental): Native WYSE CHOICES App 2^nd^ Arm (Control): Alternative app, financial literacy/budgeting 4 weeks	Female, aged 16–20 year. American Indian or Alaska Native and living in a town or city N = 439	2 Arm randomized, parallel-assigned, open-label trial Fully remote	Evaluate the effectiveness of the mHealth translation of American Indian Youth CHOICES for preventing alcohol-exposed pregnancy and fetal alcohol spectrum disorder	Alcohol and contraceptive use in the last 30 days measured at 2, 6 and 12 months	Native Wyse CHOICES app influenced changes in knowledge and behavior at one month. Trial ongoing
Luderer et al. 2022 New York, U.S. [32]	Protocol Opioid/substance use disorder	1^st^ Arm (Active comparator): Device: reSET-O 2^nd^ Arm (Experimental): Device: PEAR-008 12 weeks	Individuals with opioid use disorder, buprenorphine treatment Enrollment ongoing	Decentralized, randomized single blinded controlled trial Hybrid	Investigate interaction with DTx new content delivery format. Evaluating treatment success	Evaluate Participant Engagement Data Evaluate the number of active sessions per week between PEAR-008 and reSET-O	Not yet available
Rosa et al. 2015 U.S. [1]	Review E-technologies in clinical trials exemplified with opioid/substance use disorder	Not applicable	Not applicable	Literature review	Provide a general overview of the use of e-technologies in clinical trials research	Not applicable	Potential of e-technologies to reach a wide audience, making trials more efficient, reducing costs
Loebenberg et al. 2023 United Kingdom [68]	Case study of a remote trial Alcohol abuse/ Detecting & managing participant deception	1^st^ Arm: Drink Less app 2^nd^ Arm: National health service Alcohol advise web page to reduce drinking 6 months	Adults (18+) with hazardous/harmful alcohol consumption N = 5602	2 Arm parallel-assigned, randomized trial Fully remote	Evaluating the effectiveness of an alcohol reduction app, Drink Less. Highlight and discuss the issues with participant deception affecting remote research trials with financial compensation; and the importance of rigorous data management to detect and address these issues	Change between baseline and 6‐month follow‐up in self‐reported weekly alcohol consumption estimated over the last month. Number of participant deception	Of the 1142 participants who enrolled in the first 2 months of recruitment, 75.6% (n = 863) of them were identified as bots during data screening. Manual participant deception occurred throughout the study. Of the 5956 participants (excluding bots) who enrolled in the study, 298 (5%) were identified as false participants
Glass et al. 2023 Washington, U.S. [40]	Protocol Substance use disorder	1^st^ Arm (Active Comparator): Standard implementation reset & reset-O 2^nd^ Arm (Experimental): Standard implementation + health coaching 3^rd^ Arm (Experimental): Standard implementation + practice facilitation 4^th^ Arm (Experimental): Standard implementation + health coaching + practice facilitation 12 weeks	Primary care clinics with at least one reSET/reset-O trained clinician Adults (18+) with unhealthy substance use in primary care	4 Arm cluster-randomized, 2x2 factorial-assigned single-blind trial Hybrid	Estimate the effect of practice facilitation and health coaching implementation strategies in increasing the reach and fidelity of a DTx for substance use disorders in primary care clinics	Reach of the DTx to patients in the primary care clinic & Fidelity of patients’ use of the DTx to clinical recommendations	Not applicable
Wouters et al. 2022 Belgium [44]	Clinical trial report Cardiac monitoring, atrial fibrillation	1^st^ Arm: Device: seven-day ECG Holter & 24-hour blood pressure monitor on smartphone 2^nd^ Arm (Comparator); Device: seven-day ECG Holter & 24-hour blood pressure monitor on smartwatch 6 months	Cryptogenic stroke and transient ischemic attack (TIA) patients, older than 18 years N = 44	2 Arm double blind randomized parallel assignment clinical trial Fully remote	Demonstrate the added value of mHealth to detect atrial fibrillation (AF) early in (TIA) patients	AF detection with mHealth versus ILR – Percentage Percentage of patients with AF detected	PPG-based mHealth able to detect AF in a patient in which AF was confirmed on the ICM. Many false-positive AF on ICMs registrations
Jeganathan et al. 2022 Michigan, U.S. [35]	Protocol Cardiac rehabilitation	1^st^ Arm (Experimental): Smart watch + VALENTINE App 2^nd^ Arm (Active comparator): Usual care + smart watch 6 months	Cardiac rehabilitation patients age > 18 < 75 years	2 Arm randomized-controlled, open-label trial Hybrid	Evaluate an mHealth intervention for cardiac rehabilitation	Change in 6 minutes walk distance and step count after 6 months	Not applicable
Magnani et al. 2021 U.S. [69]	Adaption report of ongoing clinical trial Atrial fibrillation	1^st^ Arm (Experimental): Intervention relational agent/AliveCor Kardia 2^nd^ Arm (Active Comparator): Usual care 4 months	Patients living in a rural area with atrial fibrillation N = 130	2 Arm randomized, parallel-assigned, single-blind trial Hybrid	Summarize trial adaptation from in-clinic to virtual design in response to the COVID-19	Successful adaptation to virtual engagement and recruitment	Demonstrated potential to virtually recruit and involve older adults in trials of digital health interventions
Pfaeffli Dale et al. 2015 New Zealand [66]	Mixed method study Ischemic heart disease	1^st^ Arm: Usual care + mHealth application 2^nd^ Arm (Control): Usual care 6 months	Ischemic heart disease patients N = 171	2 Arm Hybrid	Study: to conduct a process evaluation of the HEART intervention to determine what worked and what did not, as well as acceptability and usability. Trial: Investigate effectiveness of an intervention designed to increase physical activity and thereby exercise capacity	Process evaluation: by means of Web site usage statistics, quantitative surveys, and qualitative interview Maximal oxygen uptake, compared to usual cardiac rehabilitation care alone (control)	High usage rates supported recent evidence that older adults do not necessarily experience a digital divide. HEART intervention was well received but additional tailoring could improve the program
Lokker et al. 2021 Ontario, Canada [36]	Protocol Hypertension	1^st^ Arm (Experimental): Intervention App Sphygmo BP 2^nd^ Arm (No intervention): Educational Control Group 6 months	Adults (18+) with hypertension	2 Arm pilot randomized, parallel-assigned open-label trial Fully remote	Test the feasibility of using the web research platform to conduct efficient and rigorous online randomized controlled trials of mHealth apps relevant to patients with cardio vascular risk factors	Participation completion, Change from baseline blood pressure at 6 months	Not applicable
Bilbrey et al. 2024 Washington D.C. U.S. [57]	Clinical trial report Cardiac rehabilitation	RecoveryPlus.Health Digital cardiac rehabilitation program: telehealth coaching and mHealth app exercises No comparator 12 weeks	Adults (45+) with stable cardiovascular disease with referral to cardiac rehabilitation N = 75	Single arm, open-label, within subject design trial Fully remote	Assess feasibility and impact of a home-based cardiac rehabilitation program in patients with cardiovascular diseases	Efficacy; change in 6-minute walk test, resting heart rate, quality of life with the 12-Item Short Form Health Survey before and after 12 weeks	The home-based cardiac rehabilitation program showed feasibility and efficacy
Tunis et al. 2024 U.S. [64]	Clinical trial design report Cardiovascular disease; Heart failure	1^st^ Arm: Sensor-controlled digital game, smart cale and activity tracker 2^nd^ Arm (Control): Sensor only 24 weeks	Older adults with heart failure N = unreported	2 Arm randomized clinical trial Hybrid	Examine challenges and opportunities of digital health interventions that affect health equity. Assess efficacy of digital health tools for improving adherence to self-care behaviors in patients with heart failure	Health equity in the biological, behavioral, physical environment, socio-cultural and healthy system domain. Heart failure proximal outcomes and distal outcomes	Demonstrated strategies for digital health interventions e.g. DCTs to effectively address health equity via a framework encompassing social and digital determinants of health. Trial ongoing
Braley et al. 2021 Massachusetts, U.S. [45]	Clinical trial report Speech therapy for aphasia in post-stroke patients	1^st^ Arm (Experimental): PCT 2^nd^ Arm (Active comparator): Conventional workbook therapy 10 weeks	Adults > 18years with cognitive, speech, or language disorders due to stroke N = 36	Open-label, randomized parallel assignment trial Fully remote	Assess feasibility and clinical efficacy of a virtual speech, language, and cognitive DTx	Western Aphasia Battery Revised, Aphasia Quotient (WAB-AQ)	CT-R group had WAB-AQ scores 6.43 higher than the workbook group
Kim et al. 2021 Canada [37]	Protocol Aphasia	1^st^ Arm (Experimental): VoiceAdapt Intervention 2^nd^ Arm (Control): No intervention 13 weeks	Patients with post-stroke aphasia age > 18 years	2 Arm randomized, waitlist-controlled, crossover single-blind trial Hybrid	Examine impact of adaptive speech-language treatment app	Change in naming on the Boston Naming Test	Not applicable
Lei et al. 2025 Missouri, U.S. [58]	Clinical trial report Stroke/ post-stroke functioning	1^st^ Arm: interactive self-management augmented by rehabilitation technologies (iSMART) intervention, technology-based skill-building education, human coaching & text messaging 2^nd^ Arm (Control): Standard of Care, Post-stroke information 12 weeks	Adults (18+) 3months post ischemic or hemorrhagic stroke with mild-to-moderate severity N = 24	2 Arm randomized parallel-assigned, open-label trial Fully remote	Examine treatment satisfaction, user experience and initial effect of the iSMART intervention on post-stroke functioning	Acceptability, appropriateness, feasibility and satisfaction at 12 weeks. Self-efficacy and functioning via stroke impact scale at week 0 and 12	iSMART enhanced post-stroke functioning and supports stroke survivors managing chronic conditions
Markland et al. 2023 U.S. [38]	Protocol Urinary incontinence	1^st^ Arm (Active comparator): Myhealthebladder, mHealth education & behavioral strategies 2^nd^ Arm (Active Comparator): VA Video connect, telehealth visits 8 weeks	Women veterans (18+) with urinary incontinence	2 Arm randomized, parallel-assigned, single-blind trial Fully remote	Determine the optimal method for remote delivery of behavioral therapy for UI to women Veterans. Compare MyHealtheBladder to a best usual care model delivered by a clinician with UI expertise via virtual video	Change in International Consultation on Incontinence Questionnaire Urinary Incontinence Short Form	Not applicable
Merlot et al. 2023 Bordeaux, France [52]	Clinical trial report Endometriosis related pelvic pain	1^st^ Arm (Experimental): Endocare consisting of visual and auditory therapeutic procedures 2^nd^ Arm (Active comparator): Digital Control 5 days	Women (18+) with endometriosis N = 102	2 Arm randomized, parallel-assigned, sham-controlled, full blind trial Fully remote	Evaluate the effects of Endocare compared to a digital control on the mean change in pain intensity 1, 2, and 3 hours after daily use during the 5 most painful consecutive days of the month in women with chronic pelvic-perineal pain	Pain intensity change from before to after treatment during 5 days	Feasibility and effectiveness of VR software use without medical supervision (at home). The decrease of analgesic use and of pain intensity provides evidence for the integration of VR in multimodal chronic pelvic pain treatment strategies
Weinstein et al. 2022 U.S. [54]	Clinical trial report (of design report below Weinstein et al. 2021) Stress urinary incontinence	1^st^ Arm: Leva Pelvic Digital Health System 2^nd^ Arm: Kegel pelvic floor muscle exercises 8 weeks	Adults (18+) with self-reported stress urinary incontinence N = 363	2 Arm randomized, controlled, double-blinded trial Fully remote	Evaluate whether pelvic floor muscle training using a motion-based digital intravaginal device is more effective than home pelvic floor muscle training for treatment of stress or stress-predominant mixed urinary incontinence	Urogenital Distress Inventory, Bladder diaries evaluating the average number of stress-incontinence episodes in 3 days	The median number of SUI episodes on the 3-day bladder diary was significantly reduced in the intervention group compared with control group, respectively. A significantly greater number of participants in the intervention group than in the control group reported they were “much improved” or “very much improved” on the PGI-I
Weinstein et al. 2021 U.S. [63]	Clinical trial design report Stress urinary incontinence	1^st^ Arm: Leva Pelvic Digital Health System 2^nd^ Arm: Kegel pelvic floor muscle exercises 8 weeks	Adults (18+) with self-reported stress urinary incontinence N = 363	2 Arm randomized, controlled, double-blinded trial Fully remote	Evaluate whether pelvic floor muscle training using a motion-based digital intravaginal device is more effective than home pelvic floor muscle training for treatment of stress or stress-predominant mixed urinary incontinence	Urogenital Distress Inventory, Bladder diaries evaluating the average number of stress-incontinence episodes in 3 days	Not applicable
Vilela et al. 2024 Brazil [59]	Clinical trial report Stress urinary incontinence	1^st^ Arm: My Pelvic Floor mobile app 2^nd^ Arm: Booklet 12 weeks	Women (18+) with stress urinary and/ or mixed incontinence N = 104	2 Arm randomized, controlled, double-blinded trial Fully remote	Verify the effects of using a mobile app on pelvic floor muscle training in women with stress urinary incontinence	Incontinence Questionnaire-Short Form and the King’s Health Questionnaire	Pelvic floor muscle training reduced symptoms via the use of the mobile app and the booklet (both groups)
Christoforou et al. 2017 London, United Kingdom [50]	Clinical trial report Agoraphobia	1^st^ Arm (Interventional): Agoraphobia Free, provides an interactive game-based intervention using cognitive behavioral techniques 2^nd^ Arm (Active Control): Generic stress and anxiety reduction app 12 weeks	Adults (18+) suffering from agoraphobia N = 170	2 Arm randomized parallel-assigned, assessor-blinded trial Fully remote	Test the clinical effectiveness of a novel mobile app for agoraphobia in a community-based sample.	Severity of agoraphobic and panic symptoms are measured using the Panic and Agoraphobia Scale at baseline, 6 and 12 weeks.	Participants who received Agoraphobia Free did not improve more than those who received the Stress Free app. Both groups showed reductions in statistically significant symptom severity. Attrition was high
Catella et al. 2022 U.S. [49]	Clinical trial report Fibromyalgia	1^st^ Arm (Active comparator): Digital Acceptance and Commitment Therapy (ACT) 2nd Arm (Active Comparator): Digital Symptom Tracker 12 weeks	Fibromyalgia patients aged 22–75 N = 67	2 Arm randomized, active controlled, phase 2 open-label, pilot trial Hybrid	Feasibility of a predominantly virtual clinical trial in an FM population and preliminary evidence for the safety and efficacy of a digital ACT program for FM (FM-ACT)	Mean change of Revised Fibromyalgia Impact Questionnaire Total Score from baseline to 12 weeks	FM-ACT demonstrated improved outcomes compared to FM-ST, with high engagement and low attrition in both arms
Bischof et al. 2022 Lübeck, Germany [33]	Protocol Internet use disorder	1^st^ Arm (Intervention): Stepped-care approach (app, telephone counseling, online therapy 2^nd^ Arm (Control): Placebo, prevention module in app 17 weeks	Participants between 16–67years	2 Arm randomized, observer-blind, parallel group trial Fully remote	Evaluation of a comprehensive and economic care system based on a stepped care approach for internet use disorder	Number of criteria for problematic internet use adapted to the gaming disorder criteria in DSM-5 and ICD-11 after 6 months	Not applicable
Donnelly et al. 2018 Dublin, Ireland [65]	Mixed method study (Clinical trial report + qualitative Study) Balance disorders and other conditions related to high risk of falls	A falls prevention program in a nursing home with supporting technological solutions No comparator 8 weeks	Nursing home staff & residents Staff (N = 10) Residents (N = 11)	Participatory design approach, 8 weeks trial, semi-structured interviews, qualitative study Hybrid	Explore burden associated with a remote trial in a nursing home setting on both staff & residents	Live experience/ burden associated to remote trial participation	Staff reported extensive burden to support activities of the trial. Residents less burden
Hunt et al. 2023 U.S. [67]	Review Asthma	Not applicable	Asthma patients	Not applicable	Report examples of remote asthma clinical studies, explore the benefits and potential drawbacks of virtual clinical investigation, potential impact on equity and representation in asthma research, provide suggestions for DCT implementation	Not applicable	Implementing DCTs using mHealth technology is a feasible and encouraged alternative to traditional in-person clinical asthma studies and can enhance equity in research representation
Thorndike et al. 2021 U.S. [41]	Protocol Chronic insomnia	1^st^ Arm (Experimental): DTx Somryst PEAR-003A No comparator 9 weeks	Patients with chronic insomnia aged 22–75	Single group assigned, open-label trial Fully remote	Assess the potential benefits of treating insomnia with an asynchronous, mobile, tailored prescription DTx	Change in Insomnia Severity Index Evaluate PEAR-003A Engagement and Adherence Data	Not applicable
Berube et al. 2024 New York, U.S. [43]	Protocol Typ 2 Diabetes	1^st^ Arm: Personalized Guidance, DayTwo app 2^nd^ Arm: Standardized 3^rd^ Arm: Usual Care Control 6 months	Adults (21+) with early stage Typ 2 Diabetes, three months average blood glucose level, hemoglobin (HbA1c)<8% with metformin use	Parallel, randomized, single-blind, three arm trial Fully remote	Determine the efficacy of a personalized behavioral approach for dietary management of type 2 diabetes, vs. a standardized behavioral intervention	Mean Amplitude of Glycemic Excursion and HbA1c Levels after 3 and 6 months	Not applicable

## Results

Fig 1 represents the selection process of sources scanned and included for this review. In total 335 articles were screened and 41 included by two reviewers. Among the included articles are thirteen study protocols [31–43], eighteen clinical trials [44–61], three clinical trial design reports [62–64], two mixed method studies [65,66], two reviews [1,67] and three articles discussing a specific part or adaption of a clinical trial [68–71].

### Characteristics of sources

A summary of references, type of the article and targeted disease/condition, intervention types, study populations, the methodologies used, the aim of the studies, the outcome measures, and other important results of all the articles included in this scoping review are presented in Table 1 below.

### Synthesized results from Table 1

A total of 41 articles were reviewed with 29 originating from the U.S., 7 from Europe, 2 from Canada, 1 from Brazil, 1 from Japan and 1 from New Zealand. All studies were published between 2015 and 2025. Types of DTx tested or discussed were mainly targeting mental health (12/41) [53,70] out of which 10 specifically focused on depression [31,34,42,46–48,51,55,56,61], or substance use disorders (7/41) [1,32,39,40,60,62]. Other targeted conditions were cardiovascular diseases (7/41) [35,36,44,57,64,66,69], stroke/ aphasia (3/41) [37,45,58] and pelvic floor issues such as urinary incontinence (5/41) [38,52,54,59,63], illustrated in Fig 2. The studies discussing a trial varied in the design regarding arms, blinding, type of control/comparator, duration, sample size and population. Most trials (34/38) included a control group, and the number of arms ranged between 1 and 16. Trials with multiple arms used randomization techniques. Open-label, single and double blinding was used, and trial phases were described as either feasibility pilot study, early-phase or phase 3 trial. The duration of the intervention varied from 3 hours to 6 months and the sample size ranged between 11 and 5602 participants. Interventions were either a DTx or a SaMD in form of mobile apps or combined with wearable devices, medical devices or telehealth. Studies differed in terms of the DCT elements employed. Some only decentralized the delivery of the intervention; others mixed centralized and decentralized elements (hybrid) such as in the recruitment process and some were fully decentralized. Although aims of the studies differed, the majority of them evaluated the efficacy or feasibility of the DTx. Due to the diversity of DTx interventions in the trials, outcomes assessed, and important results varied across most of the studies.

### Therapeutic approaches per disease group

Therapeutic approaches used in the DTx are analyzed by disease groups to address the heterogeneity of the studies.

#### Mental illness.

All included studies treating depression [31,34,42,46–48,51,55,56,61] and mental illness [53,70] used mHealth applications and the majority of studies were using CBT elements as an intervention. The study of Akechi et al. [61] specifically analyzed the effectiveness of different smartphone-based CBT components and their order in treating depressive symptoms.

#### Substance use disorders.

The articles investigating a DTx for substance use disorders [1,32,39,40,60,62,68] were mainly using parts of behavioral therapy. The intervention Kaufman et al. [60,62] provided, was in form of a culturally tailored prevention app with educational content. Two clinical trials will compare the effectiveness of DTx when applying different implementation strategies. Park et al. [39] will investigate different levels of human touch (self-monitored, peer supported and clinically integrated) while Glass et al. [40] will test the introduction of the DTx for health care professionals (HCPs), or health coaching for the DTx on a participant level.

#### Pelvic floor issues.

Merlot et al. [52] tested a class 1 medical device, with virtual reality that could be used at home to reduce pain caused by endometriosis. Two studies [38,59] tested an mHealth solution, and Weinstein et al. [54,63] a motion-based DTx device to perform pelvic floor muscle training to improve urinary incontinence. The mHealth solution delivered educational content, behavioral change reinforcement and self-monitoring.

#### Cardiovascular diseases.

The articles focusing on cardiovascular diseases [35,36,44,57,64,66,69] were heterogeneous in terms of designed interventions. Wouters et al. [44] conducted a comparative study between a DTx and a smartwatch for detection of AF. Jeganathan et al. [35] designed a virtual cardiac rehabilitation program that included contextually tailored notifications promoting low-level physical activity, exercise tracking, goal setting through the mobile study application and weekly activity summaries via email. A similar study was conducted by Bilbrey et al. [57]. They tested a remotely delivered guideline-concordant cardiac rehabilitation intervention that combined synchronous telehealth exercise training via videoconferencing and asynchronous coaching through a mHealth app, supported by remote vital sign monitoring using Bluetooth-enabled sensors. In Lokker et al.’s [36] study, a web-based platform should serve as a tool to test mHealth applications in the area of cardiovascular risk factors with web-based RCTs. Tunis et al. [64] used a digital health equity framework to analyze challenges and opportunities in digital health interventions for heart failure self-care, drawing on their experience from a DCT involving multiple sensors and apps to support behavior adherence, and offering recommendations to promote health equity in future research and practice. The intervention of Magnani et al. [69] included a virtual agent that helped to decrease the feeling of social isolation, a digital health application for education, monitoring, and problem-solving, specifically developed for AF. Pfaeffli Dale et al. [66] designed an intervention that consisted of theory-based exercises and behavioral change text messages.

#### Stroke/aphasia.

Braley et al. [45] and Kim et al. [37] conducted a DCT with a DTx for speech therapy in patients with aphasia. The DTx in Braley et al.’s [45] trial delivered structured virtual cognitive, speech and language therapy that would usually be given by a therapist. Kim et al. [37] will test an app to improve lexical retrieval which is aiming to strengthen naming abilities through app-based interactions. A broader DCT was conducted by Lei et al. [58] targeting post-stroke rehabilitation. They used a remote technology-based self-management program combining skill-building education, human coaching, and interactive text messaging to improve post-stroke functioning.

#### Others (mixed diseases).

Donelly et al. [65] analyzed the burden of a DCT on participants and staff testing a fall prevention program in a nursing home. Bischof et al. [33] investigated a stepped care approach consisting of app use, telephone counselling and online therapy for internet use disorder. Catella et al. [49] conducted an acceptance and commitment therapy DCT for people living with fibromyalgia. A DTx incorporating CBT for insomnia treatment will be investigated by Thorndike et al. [41] within a real-world evidence setting. Christoforou et al. [50] included in the treatment of agoraphobia a CBT approach with individual goal setting. Hunt et al. [67] reviewed decentralized implementation of mHealth interventions in asthma research and provided a simple framework for it. The study of Berube et al. [43] will enroll adults with type 2 diabetes to evaluate the effects of three dietary counseling approaches—personalized, standardized, and usual care control—over six months, using virtual sessions, a mobile app, and sensor-based glucose monitoring, with all participants receiving mediterranean diet guidance and behavioral support.

### Challenges

Challenges and benefits were coded in Atlas.ti across all included articles, then extracted, synthesized to avoid redundancies, and compiled into Table 2. One potential problem discussed by Pratap et al. [46] in a DCT with a DTx or SaMD that requires downloading the software on a personal device is that people sometimes share a mobile phone. This raises concerns about data privacy or may act as a barrier to seeking treatment or participating in a trial due to the fear of being stigmatized. Braley et al. [45] highlighted the elevated technological support that was requested in such trial designs. Beyond the increased need for support, the DCT with AF detection reported technical difficulties such as unstable Bluetooth connection, insufficient signal and low data quality [44]. Limited data storage capacity was emphasized as another technical issue. Participants in Bilbrey et al.’s trial [57] encountered technical difficulties using an iPad paired with wireless monitors, largely due to limited digital literacy. Fraudulent behavior such as fake or double enrollment was another challenge faced in these trials [46,68]. Rosa et al. [1] underlined a risk of lacking digital health literacy among participants and the difficulty to verify their identities during the trial. Another notable challenge is the lack of guidance from the regulatory side which could hinder the widespread adoption of these types of trial [67]. Donnelly et al. [65] identified various issues in a DCT involving a DTx that were experienced by staff and residents. Comprehension, time, communication, emotional and cognitive load, engagement, logistical aspects and product accountability were perceived burdens from staff. Participants reported problems of comprehension, adherence, emotional load and interference with their personal space. Safety has been mentioned as a challenge in the literature about DCTs. It has been addressed in some of the analyzed trials but was not reported as a key issue. The most frequent measure to ensure safety was the exclusion of participants with suicidal thoughts during the screening [34,47,49,51]. As in classical trials, adverse events were documented in the DCTs and were classified by physicians. Some trials integrated emergency resources in the applications or used telemonitoring to increase safety [32]. One trial had participants who reported feeling reliant on the application [55] which was described as a potential risk in the literature about DTx [2].

**Table 2:** Summary of reported benefits and challenges in DCTs.

Benefits	Challenges
Faster recruitment	Adherence/engagement/attrition
Improved reach and access to trial and treatment (E.g. bridge waiting times)	Ensure safety of participants
Diversified participant sample (Including minorities, underrepresented groups)	Increased burden for staff or caregiver (E.g. comprehension, time or logistical restraints)
Less burden for participants (E.g. fewer administrative tasks)	Participant burden (E.g. comprehension or emotional load)
Easy data collection (E.g. passive data collection)	Organizing all assessments remotely
Better generalizability as studies were closer to real-world conditions	Data privacy, security and accuracy (Response bias due to self-reported data)
Lower costs than traditional trials	Technical difficulties/ insufficient digital literacy
Due to technology, content can be adapted to participants	Blinding of participants
Potential to alleviate social isolation	Potential to increase social isolation
Effectiveness of DTx interventions	Fraud in enrollment

Several articles reported high attrition or low engagement as the most common issues, exemplified with retention rates as low as 26% in some studies [44,50–57,59,65,67,70]. Pratap et al. [46] encountered a significant drop-out rate of 50% from the first to the fourth week with an earlier drop-out of 2.5 weeks for the minority group compared to the control group. The problem of low adherence started already with downloading the app. In the study of Arean et al. [47] 60% of the participants never installed the app on their device. A significant decline in engagement was observed after just two weeks, along with deviations from the recommended usage outlined in the instructional video. McCloud et al. [55] also pointed out a discrepancy between participants’ high self-reported engagement and actual usage, which was in reality around 30% lower. The use of the device for urine incontinency in Weinstein et al.’s study [54] dropped to 35%, already on day five. For patients in the study of Wouters et al. [44], engagement lasted longer but also experienced a clear deterioration after one month. Donnelly et al. [65] outlined non-adherence among staff and participant being attributing it to a lack of understanding of the trial and the devices used. After conducting attrition interviews, Haun et al. [70] found that participants desired more in-person interaction, as most studies primarily involved contact limited to technical support or study-related questions. The delay in receiving the intervention was suspected to be another potential factor contributing to the low adherence of the DTx usage [46]. To achieve a highly engaged and adherent sample, participants in Weinstein et al.’s trial [54] had to fill in a bladder diary for 3 days, excluding those from the trial who failed to complete this task. Furman et al. [34] and Thorndike et al. [41] required the completion of questionnaires before participants could engage with the application, as a strategy to foster adherence and engagement. Other methods applied to improve engagement or adherence were regular reminders, gamification, reward functions, monetary incentives and check-in features [32,34,38,44,46,47,54,58,61,63,64]. In contrast to most other trials analyzed, Catella et al. [49] had high engagement and adherence to recommended use in both arms of their study, with 92% and 93%, respectively.

### Benefits

The coding analysis revealed important benefits associated with DCTs using DTx. DCT and DTx enhanced the reach and inclusion of minorities like Hispanics [46] or underrepresented groups such as people living in a nursing home [65], thereby facilitating access to both treatment and research [1,32,46,57]. Three studies [47,48,57] achieved a much better representation of the population in their sample than is typical, and Pratap et al. [46] recruited participants much faster than in person. Nevertheless, it brings along the problem of a biased sample if this is the sole strategy of recruiting participants [1]. The use of mHealth, allowed for passive data collection with minimal effort from participants. Beyond recruitment, improved efficiency in screening was cited as a benefit, by means of reaching a high number of participants that could be contacted in a quick way [1]. Cost savings are also anticipated, with Anguera et al. [48] expecting a cost reduction around 50% compared to standardized procedure. This may enable faster testing of new interventions and providing safe and effective treatments on the market, easily accessible for patients. Braley et al. [45] emphasized an ease of engagement with the DTx therapy due to decreased rigid trial and treatment structures. As a fear of being stigmatized, patients do not always seek treatment or dare to participate in a trial, depending on the disease. Remote delivery of treatment by using mHealth solutions and use of technology rather than interacting with trial staff directly can reduce this barrier [1,46]. Different studies mentioned the advantage of personalizing and tailoring interventions of DTx and tasks of DCTs to participants. Another key benefit is the demonstrated effectiveness of several of the investigated DTx. Effectiveness was observed across various disease areas, with improvements in symptoms of depression, anxiety, and stress. Stroke survivors showed enhanced scores on the WAB-AQ test or post-stroke functioning, while the VR software for pelvic pain successfully reduced pain intensity and the use of analgesics. Also, participants with agoraphobia and those with fibromyalgia exhibited significant reductions in symptom severity.

### Summary and framework for DCTs investigating DTx

To our knowledge, this is the first scoping review that focuses on DCTs in combination with DTx. Even though DTx are very suitable for the conduct of DCTs [72], there is a limited number of articles that discussed or used this combination. By using a structured database search and the application of eligibility criteria, we identified 41 articles that were analyzed regarding trial rollout. Characteristics of planning, conducting, monitoring and trial design as well as important benefits and challenges were extracted through qualitative coding. Some reoccurring concepts derived from the code groups “planning”, “design” and “conduct” were e-recruitment (32/41), e-eligibility screening (30/41), e-informed consent (28/41) and the inclusion of e-PROs (36/41). Other key themes in most of the studies were the integrated reminders (24/41), passive data collection (24/41) and the patient-centered approach by using personalized treatment, feedback mechanisms, participatory design, preferences and burden assessment (25/41). The major problems encountered were high attrition, low adoption, adherence and engagement with trial activities and the DTx/SaMD (18/26). The primary advantages identified in this trial and intervention type were better access to treatment and trials, and faster recruitment than with traditional ways.

**Fig 3:**
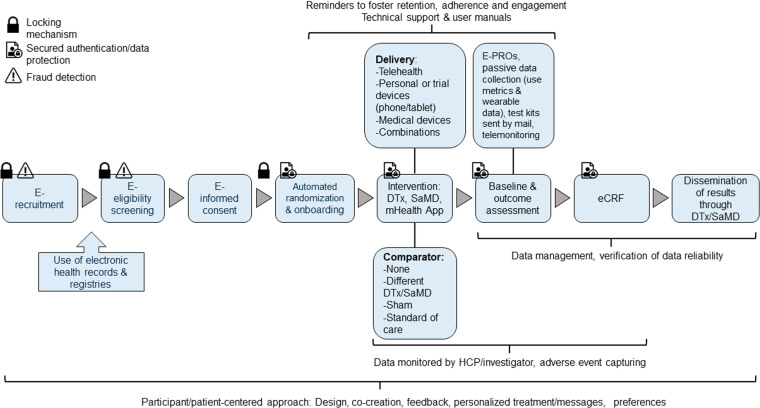
Identified design, steps and technical features of DCTs with DTx among analyzed articles. Reoccurring steps with features to integrate, to enable DCTs as safe and effective as possible. Locking mechanism: Prevents participants from altering their responses. For example, if a participant reports smoking during e-eligibility screening (an exclusion criterion), they are marked ineligible and cannot revise their answer to qualify. Similarly, the mechanism prevents re-randomization if participants are dissatisfied with their assigned treatment. Secured authentication/data protection: High data protection standards such as double authentication for log-ins into applications. Fraud detection: Mechanisms to prevent fake or double enrollment.

Identified themes and steps of the analyzed DCTs using a DTx are summarized into a framework shown in Fig 3. It is proposed to take the displayed concepts into account when designing future DCTs with DTx.

### Step-by-step guidance through the framework

#### Participant-centered design of the interventions and trials.

Before initiating the design of a DCT with a DTx, developers should involve patients in the creation of the application. Given that low DTx engagement was identified as the most significant problem in these trials, a participant-centered design of the intervention is essential to get the users to interact with the app. This participant-centered approach should also extend to the design of the DCT, to maximize convenience for the participants, to foster inclusion, prevent drop-outs and increase adherence to the trial protocol. An example is considering participants’ preferred times of the day for completing trial tasks, as stated by them in the beginning [31]. Other examples used in the analyzed studies can be found in S1 Table.

#### Recruitment, screening and inclusion of participants.

Once the DTx and the trial are designed, e-recruitment is the first step. E-recruitment methods include the use of social media platforms or even machine learning to optimize participant recruitment. Mixed approaches with traditional methods of recruitment should be considered. Screening of participants can be done either simply online (e-eligibility screening) or in combination with an interviewer over the phone. Recruitment and screening should be facilitated by electronic health records or electronic patient registries. They can help to identify potential participants which then can be specifically contacted, e.g., via email or to automate screening for eligibility as many criteria can be checked with the information in the records. In addition, self-reported screening forms can be designed to automatically exclude participants who do not meet eligibility criteria, rather than simply replicating the paper-based form in electronic format.

To hinder participation of ineligible patients, introducing a locking function for selected answers in the eligibility screening can become necessary. The same strategy can be used at a later stage in the randomized allocation of the treatment. Additionally, single-use study links for device and per user should be considered to prevent multiple enrollments by the same person. Integration of automated tools to detect participant deception and phone number verification can reduce fake enrollments.

Consent can be obtained either fully electronically or in combination with a phone call to clarify remaining questions. Besides the read-only consent information, interactive links, a video about the study, a subsequent quiz to test participants’ understanding, and follow-up contact in cases of unclarity are options to improve traditional ways of a consent procedure. Additional confidentiality agreements might need to be signed, for example if the clinical trial includes telemedical group discussions. An important aspect is to check whether the electronic signature is accepted or not in the country where participants are recruited. Randomization can be automated via a computer randomization tool.

Either before or after randomization, a step referred to as “onboarding” of participants is recommended. In the analyzed trials, the term “onboarding” was described as the process of introducing the participants to the DTx (downloading the application, pairing devices with smartphone, registering, explaining the trial protocol). Onboarding is an important step as the participants will use the intervention independently compared to a drug trial where a HCP is administering it. Methods of onboarding are instructional videos, user guides, support manuals or tutorials that support the downloading process of the application and proper utilization of the intervention or device, respectively. Sometimes staff can remotely help with downloading and explaining the use of the DTx/SaMD. Depending on the complexity of the trial, for example when DTx are used in combination with devices, in-person instructions or visualization methods prior to the start might be necessary to familiarize patients with their use. Resources, instructions and contact details for technical aspects can be included in the onboarding procedure. In some cases, smartphone training, especially for those without previous smartphone experience, can be essential.

Participants could use their own smartphone or tablet or be provided with a preconfigured device containing the application. Other trial materials like wearable devices or self-administered test kits can be shipped to the participant’s home.

#### Delivery of interventions.

Interventions can be delivered through the mHealth applications but also in combination with telemedicine with an HCP or with in-person visits. Sometimes a caregiver needs to be available to assist participants in trial activities. DTx intervention delivery can also come in conjunction with medical devices such as virtual reality glasses or devices for pelvic floor training. Categories of suitable interventions depend on the targeted disease but include for example CBT, educational content, physical activity, acceptance and commitment therapy, counselling or speech and cognitive language therapy. To control the DTx, different options are available such as standard of care, an active comparator (a different DTx), none (waitlists) or a sham DTx.

Automated reminders via email or text message or daily notifications for study participants to complete the required tasks such as engaging with the intervention or complete PRO questionnaires is indispensable. Reminders also include active calls from the study teams or mail sent to participants’ homes.

#### Outcome assessment, data collection and monitoring.

Outcome assessment is possible either through the DTx, in combination with teleconsultation, with recordings from wearables and devices or with surveys that are accessible by links that participants receive by text message, email or in the app. Although outcomes assessed with DTx are predominantly ePROs, data from health records or self-administered test kits can be used as well. Besides the data collected through PRO or other assessments, apps can simultaneously collect passive data, such as number of log-ins, mostly to track engagement with the DTx but also as a monitoring option for HCPs.

The use of electronic data capture systems and electronic case report forms (eCRF) for the trial data reduces potential errors by transferring the data from paper to the computer. To ensure the participants’ safety and data reliability, the surveillance of self-administered tests by telemonitoring can be implemented. Data entered by patients can be double checked and monitored by HCPs or researchers. Adverse events must be captured either by integrated questions in surveys, outcome assessment (remote and in-person) or by integrated message functions. Those can be reviewed by clinicians and emergency resources should be implemented in the application to guarantee the safety of participants. Electronic evaluation of data stemming from the applications by algorithms to detect device malfunctioning is an option to improve safety during the trial. Collecting reports of encountered technical problems during the trial and diaries to note experiences is a possibility to improve future trials.

#### Other steps and measures to consider.

In addition to the intervention delivery and data collection, a DTx can also serve as a platform to distribute research findings. For DCTs investigating DTx, robust measures to ensure data protection are critical, given the heightened risk of cyberattacks, data breaches, and violations in the digital domain. Recommended measures include the use of encrypted databases, cloud storage, participation verification, servers that comply with the local regulatory requirements as well as two factor authentication logins.

International guidelines such as good clinical practice and national regulations must be respected when using decentralized elements like e-consent or e-recruitment, and the trial may need to be adapted to align with these standards.

## Discussion

### Type of evidence and trends

This scoping review investigated the different steps, challenges and benefits of undertaking a DCT when investigating a DTx or SaMD and its implications for further trials. The body of existing literature is small with only 41 identified articles mainly originating from the U.S. (29/41). But with digital health as a fast-moving field, an increase in relevant published articles is to be expected, also from other geographical areas.

The scoping review identified a trend towards DTx in the area of mental health (12/41) from which 10 investigated digital health solutions for depression. Especially with the elevated psychological burden caused by the COVID-19 and the climate crisis, evidence-based digital solutions to mitigate this mental health crisis are more than needed [73,74]. DCTs testing DTx for mental health should be fostered as they can reach remote patients, help solutions to get to the market faster and lower the barrier to access treatment related to stigma [1,46,48].

### Regulatory and research gap

This review reveals a gap in regulatory considerations among the selected articles, likely stemming from limited and simple regulatory guidance [1,67]. While Europe has issued position papers on DCTs and the Food and Drug Administration released new guidance in May 2023 [20–22,75], these documents offer broad outlines rather than detailed instructions. The overlapping regulations and definitions governing DTx and SaMD, combined with regional variations in certification requirements [76], add significant complexity. Trials combining DTx with medical devices further complicates navigating in the regulatory landscape. This lack of clarity and complexity can lead to inconsistent use of terms like DTx, mHealth or SaMD and impede researchers conducting standardized research. A comprehensive regulatory framework, including tailored guidelines for aspects such as good clinical practice in DCTs and trial design requirements for combining DCTs with DTx, is essential to enhance DCTs with mHealth solutions and ensure clear evidence generation.

While engagement has been recognized as a challenge, only one of the articles provided background information on how the proposed trial time, duration or structure of engagement was defined. This lack of clarity or consideration is problematic since the engagement can be equated to the “dosage” of a DTx intervention directly influencing the clinical outcome of a trial. Therefore, to fill this research gap and acknowledge this important aspect, future studies should consider the 4-step framework suggested by Strauss et al. [77] to define and achieve meaningful engagement with DTx. Another related challenge is that many apps and interventions in the analyzed trials were multicomponent, making it unclear which specific element contributed to the observed effects, as only one study used a factorial trial design to evaluate individual components.

### Addressing the challenge of engagement

The most frequent challenge encountered in the analyzed studies was low engagement with the DTx and required trial tasks. For a DTx to unfold its efficacy, users must actively and sufficiently engage with it and without adherence to trial protocols, results cannot be relied on [77]. Barriers to adoption and engagement with mHealth or other digital health solutions include a lack of knowledge or information about benefits of the intervention, concerns about data security and privacy, insufficient support from HCPs, low digital health literacy, complex designs of interventions or technical difficulties [78–81]. Additionally, taking a pain-killer requires less effort than doing a 20 minutes DTx intervention to relieve pain [52]. Reminders, personalization, incentives and a patient-centered approach were used to maximize engagement with the DTx and adherence to the trial protocol. However, further approaches are needed. These could involve strategies such as a better introduction to the intervention by HCPs or highlighting or increasing privacy and data protection measures [78]. This is also supported by the suggestion of Pfaeffli Dale et al. [66] who propose to involve clinicians, for example with goal setting, monitoring progress, providing inputs or follow-up appointments which could keep participants accountable and therefore more engaged. Results from two of the analyzed protocols [39,40] will potentially clarify whether such implementation strategies could address the engagement problem of DCTs with DTx. Moreover, Rothman et al. [42] emphasized the importance of developing a standardized metric for adherence to digital interventions to enable more meaningful comparisons across studies. It is important to note that a general hybrid approach does not necessarily resolve the attrition and engagement problem. Both, fully remote and hybrid trials analyzed in this review, suffered from this problem. It is essential to explore whether the challenges in participant engagement are more closely linked to the specific characteristics of the clinical trials—such as being experimental and decentralized—rather than to the digital health solutions themselves. The anticipated benefit of high engagement in DCTs involving DTx, which is expected due to reduced participant burden [3,16], may be outweighed by factors such as limited interaction with HCPs and peers, or perceived inferiority compared to already approved DTx. Future studies should investigate this issue, for example, by conducting interviews with participants who exhibited low engagement in these trials.

### Practical implications for future research

Although the trials showed a big heterogeneity in terms of design (number of arms, duration, participants, blinding) and investigated areas of health, reoccurring terms and concepts have led to the development of a framework for DCTs testing DTx. To augment standardization of DCTs investigating DTx, we suggest considering the conceptual framework displayed in Fig 3 as practical implication for future research. Nevertheless, it is crucial to consider certain points in relation to the different steps. For example, the integration of e-recruitment and e-eligibility screening techniques can bring the advantage of faster and cheaper recruitment but at the same time have the potential of introducing a recruitment bias (not representative/diverse enough sample) [82]. This has been observed in DCTs with DTx included in this scoping review [49,56]. Another critical point to consider is fake enrollments. Automated tools or not mentioning financial compensation during e-recruitment has been suggested to tackle this problem [68]. The majority of articles included used as well e-consent options. Skelton et al. [83] found in their systematic review that e-consent is well-adopted by patients, study comprehension was high and user-friendly applications were available. Nonetheless, they stress offering paper-based versions to respect patient preferences and highlight the importance of data protection. PROs or biomarkers coming from data gathered via passive data collection or wearable devices integrated in the trial are especially suitable for outcome assessment of DTx in a DCT. These options facilitate easy data collection but ethic committees expressed concerns of data and scientific reliability [84,85]. Another concern is the provision of the participants safety and addressing the additional technical hurdles when conducting a DCT [11,16,32,85]. The control of possible addiction [2] the monitoring of adverse events and unintended use of DTx should be taken into account as interaction with apps of some groups remain understudied [46]. However, only two adverse events related to DTx use have been reported in the analyzed reports. Although digital (health) literacy, mobile phone subscribers and access to internet are constantly increasing, the “digital divide” in the use of technology for clinical trials and treatment delivery, has to be acknowledged and strategies developed to overcome the gap [2,4,86]. For example, the Digital Health Equity Framework proposed by Richardson et al. [87], along with the recommendations by Tunis et al. [64] for addressing health equity in digital interventions, could be incorporated when designing a DCT using a DTx. On a positive note, in the trial conducted by Braley et al. [45], most participants were older than 80 years and managed their devices and its use without worth mentioning problems. A shift of burden towards the participants in DCTs [65] was also observed by ethics committee members, who are an important part when planning a clinical trial [84]. Researchers should provide increased support for participants, for example in form of videos, assistance buttons or user manuals to solve technical difficulties related to the trial tasks or the use of the DTx such as it was done in some of the analyzed articles.

Even though hypothetically all the steps of a DTx trial could be conducted in a decentralized manner, not every trial is suitable nor are always the means available to provide everything fully decentralized. Therefore, it is recommended to adapt every trial to the context. This aligns with previous findings from DCTs [3]. Sometimes even flexibility between participants should be considered due to differences in digital health competencies or preferences. These approaches aim to contribute to overcoming the current challenges in engagement.

## Limitations

Although this is the first scoping review that analyzes DCTs with DTx, the four databases might not cover all articles as the literature of digital health is rapidly evolving. Besides that, a valuable insight would have been an analysis regarding the quality of the included articles or the exchange with stakeholders as suggested by Arksey and O’Malley [25]. Nevertheless, with our in-depth analysis of the articles included we were able to provide a framework with step-by-step guidance for this type of trial, which should contribute to improving the quality of those studies and guide future research. The provided framework is conceptual and did not investigate best practices or build recommendations for detailed methodological aspects such as trial duration, blinding strategies, and control conditions tailored to the specific condition and intervention under study. Defining these elements for each disease category or type of DTx intervention could be a key next step in standardizing this trial design.

## Conclusions

This scoping review of DCTs evaluating DTx provides a comprehensive framework to guide future research and standardize this trial design. The framework and the step-by-step guidance should help researchers avoid common pitfalls, identify key areas requiring attention, and determine whether a DCT is the appropriate approach for evaluating a DTx. This work found that existing DCTs assessing DTx have demonstrated safety, yielded meaningful results, and effectively reached and included participant. The expected and often claimed benefit of having better retention rates in DCTs, could not be observed in the conducted DCTs with DTx. Moreover, despite participant-centered designs and co-creation of the trials and DTx interventions, engagement with the DTx was significantly low. Further investigations into retention, adherence and engagement in DCTs with DTx will be essential. Specifically, it remains to be examined whether low engagement with DTx persists outside of the controlled clinical trial setting such as in a routine healthcare setting. This emphasizes the need for real-world evidence studies besides traditional RCTs as already stated by other researchers in the field [6].

## Supporting information

S1 File Protocol for the Assessment of Digital Therapeutics in Decentralized Clinical Trials: A Scoping Review. (DOCX)

S2 File Preferred Reporting Items for Systematic reviews and Meta-Analyses extension for Scoping Reviews (PRISMA-ScR) Checklist. (DOCX)

S1 Table Detailed measures and themes derived from the qualitative code groups “planning”, “design” and “conduct” of DCTs. (DOCX)
