# Supplementary material for: Assessment of digital therapeutics in decentralized clinical trials: A scoping review
Source: PLOS Digit Health. 2025 Jun 23;4(6):e0000905. doi: 10.1371/journal.pdig.0000905 (PMC12185025; doi:10.1371/journal.pdig.0000905)
Supplement: S1 File — (DOCX) [file pdig.0000905.s001.docx]

**S1 File. Protocol** **for the Assessment of Digital Therapeutics in Decentralized Clinical Trials: A Scoping Review.**

Cinja Koller*, Marc Blanchard, Thomas Hügle

Department of Rheumatology, University Hospital Lausanne (CHUV) and University of Lausanne, Switzerland

*Corresponding author
Contact: Cinja Nadana Koller, MSc
Department of Rheumatology, University Hospital Lausanne
Avenue Pierre-Decker 4, Hôpital orthopédique
Lausanne, CH 1011

[Cinja.koller@unil.ch](mailto:Cinja.koller@unil.ch)
[Cinja.koller@chuv.ch](mailto:Cinja.koller@chuv.ch)
 +41774117865

| Section and topic | Item No | Checklist item |
| --- | --- | --- |
| ADMINISTRATIVE INFORMATION | | |
| Title: |  |  |
| Identification | 1a | Protocol for the Assessment of Digital Therapeutics in Decentralized Clinical Trials: A Scoping Review |
| Registration | 2 | N/A, the protocol will be made available under supplementary material. |
| Authors: |  |  |
| Contact | 3a | Cinja Koller*, Department of Rheumatology, University Hospital Lausanne (CHUV) and University of Lausanne, Switzerland, cinja.koller@unil.ch  Marc Blanchard, Department of Rheumatology, University Hospital Lausanne (CHUV) and University of Lausanne, Switzerland, marc.blanchard@unil.ch  Thomas Hügle, Department of Rheumatology, University Hospital Lausanne (CHUV) and University of Lausanne, Switzerland, [thomas.hugle@chuv.ch](mailto:thomas.hugle@chuv.ch), *corresponding author: Department of Rheumatology, University Hospital Lausanne, Avenue Pierre-Decker 4, Hôpital orthopédique, Lausanne, CH 1011 |
| Contributions | 3b | CK drafted the protocol. MB and TH took part in the discussion of key points in the protocol. CK, MB and TH reviewed the protocol. CK is the guarantor of the review. |
| Amendments | 4 | N/A |
| Support: |  |  |
| Sources | 5a | N/A |
| Sponsor | 5b | N/A |
| Role of sponsor or funder | 5c | N/A |
| INTRODUCTION | | |
| Rationale | 6 | The COVID-19 pandemic accelerated the use of digital technologies in healthcare in general and provoked a shift in the way clinical trials are conducted. Major changes in clinical trials are reflected in increased online recruitment, adoption of electronical consent, the shipment of samples or devices to and from the patients home and telemedicine consultations. Expected benefits of these decentralized clinical trials are improved adherence due to the lower burden on participants and data which is much closer to the real-world. However, not all clinical trials are suitable to be conducted decentralized and evidence regarding the suggested benefits has not been conclusive yet. For example, clinical trials to investigate DTx fit well into the concept of DCTs as they already provide the user interface for the collection of PROs and the therapeutic intervention. DTx require fewer interaction with healthcare providers and do not bring along complex supply chain management issues. Moreover, DTx need to be investigated, optimally with a randomized clinical trial due to their definition as: “… evidence-based therapeutic interventions that are driven by high quality software programs to treat, manage, or prevent a disease or disorder. They are used independently or in concert with medications, devices, or other therapies to optimize patient care and health outcomes.”.  DCT and DTx are both facilitating access by bringing the research or the treatment to the patients home rather than the other way around. Lastly, conduct of DTx trials in a remote setting seems to be feasible in terms of safety, as the risk of serious adverse events is expected to be minimal compared to pharmacology interventions. Various countries and entities have issued recommendations and guidance on DCTs, but none specifically tailored for DTx. Additionally, no systematic or scoping review on DCT with DTx has been found, prompting the need for this review to fill a research gap. |
| Objectives | 7 | Research Question: “What insights do existing literature and practice provide regarding the application of DCT methods in evaluating DTx/SaMD?”  Objectives:   - Address the key aspects and practical considerations when planning, designing, conducting or monitoring DCTs with DTx or SaMD - Identify the type of challenges faced during these trials - Identify benefits compared to traditional clinical trials - Identify research gaps, the type of evidence currently available, trends, key terms or concepts used in the literature |
| METHODS | | |
| Eligibility criteria | 8 | Population: Patients (human) without any restrictions. Intervention or Comparator: DTx or SaMD independent of regulatory approval, mobile health application (mHealth). Study design: Interventional (Randomized clinical trial) or any type of study design reporting about experiences, learnings etc. of decentralized clinical trials with DTx/SaMD. Setting: Decentralized clinical trial. Time frame: N/a.  Inclusion criteria:   - Fully or partially decentralized clinical trials investigating DTx / SaMDs, mHealth with intention to treat, manage or prevent a disease as per definition of the DTx Alliance, unrelated to planned regulatory approval - Review articles or protocols of DCT testing DTx/SaMD - Articles reporting experiences related to the DCT or DTx under investigation   Exclusion criteria:   - Articles that are not focusing on the design, planning, conduct or monitoring or its related learnings (difficulties, benefits and advantages compared to classical trials) of the DCT - DTx/SaMD of interest is a wellness or well-being app - Device that is not focused on the treatment, management or prevention of a disease - Software in question does not have a specific medical purpose, but instead, a more general health advice-oriented approach.   No language or year of publication restriction will be used. Only peer-reviewed articles will be included. |
| Information sources | 9 | The databases Web of Science, Medline, Embase and Cochrane will be searched with a structured search strategy for the selection of peer-reviewed articles. In addition, trial registries will be used for the data extraction. Other sources such as website from stakeholders will be scanned for grey literature such as blogs or whitepapers. They will only be considered for the discussion of results and not for the inclusion of articles. Planned dates for the search are January and February 2024. |
| Search strategy | 10 | Search strategy used in EMBASE:  ((DCT OR DCTs OR VCT OR VCTs OR (trial* NEAR/3 (decentrali* OR de-centrali* OR remote* OR virtual* OR web-based OR ehealth OR end-to-end OR hybrid))):ab,ti,kw) AND ('mobile health application'/exp OR ("digital therapeutic*" OR DTx OR SAMD* OR (software NEAR/5 "medical device*") OR Mhealth OR DIGA OR "digital health application*" OR "mobile health" OR ("mobile app*" NEAR/3 treat*)):ab,ti,kw)  Filters planned to apply: No restrictions are planned. |
| Study records: |  |  |
| Data management | 11a | After applying the systematic search strategy to the different databases, the citations will be exported into EndNote. Then duplicates will be removed by the automatic tool Deduklick. Afterwards the results will be imported into the Rayyan and each reviewer will independently screen and document the reasons for exclusion via the software Rayyan. Data extraction will be done by two reviewers but not independently. The citation manager Zotero will be used throughout the whole process. |
| Selection process | 11b | After applying the search strategy to the four chosen databases, duplicates will be removed and the remaining articles will be extracted for eligibility screening. Articles will be undergoing bibliography screening and will firstly be included or excluded based on abstract screening by two reviewers. Afterwards full text screening will be done by two reviewers using beforehand defined eligibility criteria. In case of disagreement or uncertainty the reviewers will find a solution by discussion. |
| Data collection process | 11c | Data extraction will follow by two reviewers based on the articles and the information provided in trials registries. Extracted data will be gathered synchronously in a shared MS excel spreadsheet. Once the data extraction is completed the data will be reviewed independently by 2 reviewers. Disagreement or uncertainty will be resolved by discussion. |
| Data items | 12 | References, intervention types, focus of the article, study populations, the aim of the studies, the methodologies used, the outcome measures and other important results will be summarized in an MS excel spreadsheet. |
| Outcomes and prioritization |  | Firstly, on a macrolevel the following outcomes will be taken and compared:  MS Excel spreadsheet outcomes:  -References (**Author(s), year of publication, study location, citation)** -Intervention types (**Intervention type, and comparator (if any); duration of the intervention)** -Focus of the article (**Type of article, therapeutic indication)** -Study populations (**Study population characteristics, Number of participants)** -Methodologies used (**Fully remote or hybrid trial)** -Outcome measures -Important results **(related to the outcomes measured and others)**  Secondly, on a microlevel the following outcomes will be analyzed and then summarized in groups which will be made per focus of the article. Qualitative outcomes of coding:  -Design **(randomized, blinded, number of arms, fully remote vs. hybrid) -**Planning **(preparation of the trial, who, special aspects)** -Conduct **(recruitment, screening, consent, intervention, trial activities)** or  -Monitoring **(documentation, data management, analysis, adverse events, drop-outs)** information on DTx DCT.  -Learnings **(difficulties/problems/challenges and benefits/advantages compared to classical trials)** of the DCT. |
| Risk of bias in individual studies | 14 | As this is the protocol for a scoping review, no risk of bias assessment is planned. |
| Data synthesis | 15a | No meta-analysis will be performed as it will be a scoping review and not a systematic review. |
|  | 15b | N/A |
|  | 15c | N/A |
|  | 15d | N/A |
| Meta-bias(es) | 16 | N/A |
| Confidence in cumulative evidence | 17 | N/A |
