# Supplementary material for: Assessment of digital therapeutics in decentralized clinical trials: A scoping review
Source: PLOS Digit Health. 2025 Jun 23;4(6):e0000905. doi: 10.1371/journal.pdig.0000905 (PMC12185025; doi:10.1371/journal.pdig.0000905)
Supplement: S1 Table — (DOCX) [file pdig.0000905.s003.docx]

**S1 Table. Detailed measures and themes derived from the qualitative code groups “planning”, “design” and “conduct” of DCTs.**

| *Recruitment, screening and inclusion of participants* | |
| --- | --- |
| Pratap et al. | To hinder participation of ineligible patients, they introduced a locking function for selected answers in the eligibility screening. They used the same strategy at a later stage in the randomized allocation of the treatment.  Additionally, single-use study links for device and per user were sent to prevent multiple enrollments by the same person. They added a subsequent quiz to test participants understanding about the study and the consent. |
| Loebenberg et al. | Integrated an automated tool to detect participant deception and used phone number verification as they struggled with up to 75% fake enrollments. |
| Arean et al. | Besides the read-only consent information, they provided a video about the study. |
| McCloud et al. | They asked participants about their understanding of the trial and contacted them in case of unclarity. |
| Bischoff et al. | An additional confidentiality agreement had to be signed by participants as the clinical trial included telemedical group discussions. |
| Donnelly et al. | Support manuals were made available throughout the trial and in-person instructions prior to the start was realized. |
| Magnani et al. | Stressed the importance of smartphone training. |
| Christoforou et al. | Highlighted the fact that they did not provide any training for the trial or the application in test and technical issues were simply resolved by e-mail. |
| Rothman et al. | Dedicated call center for technical support and downloading the app. |
| *Participant-centered design of the interventions* | |
| Schweiger et al. | Considered participants preferred time during the day to complete trial tasks as stated by them in the beginning. |
| Moberg et al. | Thei application facilitated anonymous peer communication. Other key elements in the design to personalize it were interventions, which adapted the difficulty to the user’s ability, and individual goal setting at the beginning of the study. A combination of guided paths and parts where participants could choose whatever they liked, a so called ‘free buffet part’ was used. |
| Kaufman et al. | Applied a community guided research approach to reach trust and high engagement in research activities beyond a single clinical trial. |
| Jeganathan et al. | Used a participant-centered approach by tailoring messages to participants. |
| Lokker et al. | Included participants in the development of a web-based platform |
| Donelly et al. | Planned and designed the DCT as participant-friendly as possible by using a collaborative approach during the design stage. |
| Braley et al. & Kim et al. | Used a participant-centered approach by integrating adaptive levels, wishes and involving the targeted population in the design and development of the application. |
| Christoforou et al. | Included in the design of the intervention for agoraphobia treatment individual goal setting and required the participants to achieve these goals at the end of a session. Despite a participant-centered design of the intervention, participants mentioned stressful components of the application such as background music. |
| Hunt et al. | As an important aspect for the design of mHealth interventions they mentioned the provision of it in the participant’s preferred language. |
| Pratap et al. | They considered the participants app preference during the randomization. |
| Lei et al. | Individual activity goals, strategies to achieve them and monitoring of goal achievement via check-in messages. |
| Berube et al. | Use of algorithm to provide personalized feedback on participants’ diet. |
| *Outcome assessment, data collection and monitoring* | |
| Braley et al. | They used traditional paper-based trial data collection for data entry methods. |
| Luderer et al. | As a strategy to ensure the participants safety and data reliability, the surveillance of self-administered tests by telemonitoring was implemented. |
| Wouters et al. | Data entered by patients were double checked and monitored by nurses and doctors. |
| McCloud et al. | Trial had two adverse events reported, namely feeling distress caused by technical issues of the application and feeling reliant on it. |
| Weinstein et al. | Electronic evaluation of data stemming from the application was analyzed by an algorithm to detect device malfunctioning. |
| Donnelly et al. | They collected reports of encountered technical problems during the trial and diaries to note experiences. |
